# Medical Practice in Connecticut

**Published:** 1891-10

**Authors:** 


### MEDICAL PRACTICE IN CONNECTICUT.

The following neat bit of sarcasm under the above heading appeared in the *Boston Medical and Surgical Journal*, August 27, 1891 :

The *Monthly Bulletin of the Connecticut Board of Health* contains the following reply, sent to a doctor inquiring of a State official if he would be allowed to practise in Connecticut by registering his name and the college from which he graduated : "Sir : Anybody can practice medicine in Connecticut. You do not need to register; you do not need a medical diploma; you do not need to know the difference between opium and peppermint; you do not, indeed, need to know anything. You can simply come and live here, and begin to practise. The laws of Connecticut will sustain you in collecting your fees for professional services, if you render any which you choose to call such. But if you undertake to carry me or my trunk to the depot for pay, you must get a license. If you peddle matches or peanuts, you must get a license. If you collect the swill from your neighbors, to feed your pigs, you must get a license. If you want to empty your cesspool, you must get a license. But you can practice medicine in Connecticut *without a license*."

Verily, Connecticut has no need of a State Medical Examining and Licensing Board! She is able to manufacture nutmegs, sell peanuts, and commission her doctors without the aid of such nuisances as modern statutory enactments.

